# Antifungal Susceptibility Profile and Biocide Tolerance in Clinically Relevant Yeasts: Implications for Therapy and Hospital Infection Control

**DOI:** 10.1111/myc.70216

**Published:** 2026-09-04

**Authors:** Yasmim Passos Lima, Letícia Rebello Machado, Ricardo Villela Bastos, Victor Quinet de Andrade Bastos, Lucas Quinet de Andrade Bastos, André Netto Bastos, Claudio Galuppo Diniz, Vania Lúcia da Silva, Vanessa Cordeiro Dias

**Affiliations:** ^1^ Federal University of Juiz de Fora – UFJF Juiz de Fora MG Brazil; ^2^ Cortes Villela Laboratory Juiz de Fora MG Brazil; ^3^ Department of Morphology Federal University of Juiz de Fora – UFJF Juiz de Fora MG Brazil; ^4^ Department of Parasitology, Microbiology, and Immunology Federal University of Juiz de Fora – UFJF Juiz de Fora MG Brazil

**Keywords:** antifungal resistance, biocides tolerance, *Candida*, efflux pump, infection, treatment, *Trichosporon*, yeast

## Abstract

**Background:**

Opportunistic yeasts account for a substantial proportion of invasive fungal infections and are important agents of healthcare‐associated infections, especially in intensive care units (ICUs).

**Objectives:**

To describe the clinical and epidemiological characteristics of hospitalized patients with positive cultures for medically relevant yeasts and correlate these data with antifungal susceptibility, biocide tolerance and phenotypic efflux pump activity.

**Methods:**

Ninety‐four clinical isolates were analysed: 31 *Trichosporon asahii*, 39 *Candida albicans* and 24 other yeasts. Antifungal susceptibility and biocide tolerance were assessed by disk diffusion, and phenotypic efflux pump activity was evaluated using ethidium bromide. Clinical and epidemiological data were retrieved from electronic health records.

**Results:**

Most isolates (73.4%) originated from ICUs. Urine (55.3%) was the most frequent clinical specimen. Overall mortality was 62.7%, reaching 71.8% among patients with *C. albicans* . Notably, 68.1% of patients did not receive antifungal therapy after diagnosis. All *T. asahii* isolates were susceptible to azoles and resistant to caspofungin. *C. albicans* showed high susceptibility to amphotericin B (100%) and caspofungin (93%). Phenotypic efflux pump activity was observed in all evaluated yeasts. Sodium hypochlorite showed greater antifungal activity, with inhibition zones of up to 18.0 mm, while hydrogen peroxide (4.25%) and benzalkonium chloride (5%) showed minimal zones (~6 mm) for all isolates.

**Conclusions:**

Opportunistic yeast infections continue to pose a major challenge in healthcare settings. Our findings highlight the critical importance of accurate laboratory identification, ongoing surveillance of antifungal susceptibility, and effective infection prevention and control strategies, including the rational selection of biocides, to improve patient outcomes.

## 1 Introduction

Fungal infections in hospital settings constitute a significant and growing clinical concern, particularly among critically ill individuals, due to their high associated mortality and the challenges related to diagnosis and treatment [1, 2, 3]. Opportunistic yeasts represent a major proportion of these infections and are among the leading causes of healthcare‐associated invasive fungal diseases, especially in intensive care units (ICUs). The occurrence of these infections is strongly associated with risk factors such as prolonged hospitalization, broad‐spectrum antibiotic use, invasive medical devices, immunosuppression and comorbidities [2].

The increasing antifungal resistance among opportunistic yeasts represents a major global health concern, prompting the World Health Organization (WHO) to classify several clinically important yeasts, including *Candida* spp., among its priority fungal pathogens: *Candida albicans* and *Candidozyma auris* (syn. *Candida auris*) are in the critical group; *Candida tropicalis* and *Candida parapsilosis* are high priority; and *Nakaseomyces glabratus* (syn. *Candida glabrata*) and Pichia *kudriavzevii* (syn. *Candida krusei* ) are classified as high and medium priority, respectively [4, 5, 6]. In parallel, *Trichosporon* spp., particularly *Trichosporon asahii*, have emerged as significant opportunistic pathogens causing invasive infections with high morbidity and mortality, especially in immunocompromised and critically ill patients [7, 8, 9, 10].

The hospital environment plays a central role in the development of healthcare‐associated infections (HAIs) [11]. Frequently touched surfaces and instruments handled by healthcare professionals act as reservoirs for fungi and other microorganisms, promoting cross‐contamination among patients, staff and the hospital environment itself [12]. Effective environmental disinfection is therefore essential to limit persistence and transmission of opportunistic yeasts. Brazilian and European guidelines [13, 14] classify several compounds as broad‐spectrum biocides, including quaternary ammonium compounds, hydrogen peroxide and sodium hypochlorite, and establish standardized recommendations regarding concentrations and application protocols in healthcare settings.

Despite their widespread use, experimental data evaluating the tolerance of clinically relevant yeasts to disinfectants and antiseptics remain scarce. This knowledge gap limits our understanding of the environmental persistence of these microorganisms and may compromise infection control strategies.

In this setting, this study aims to characterize and compare clinical isolates of *Trichosporon asahii*, *Candida albicans* and other medically relevant yeasts obtained from hospitalized individuals by integrating clinical and epidemiological data with antifungal sensibility profiles, tolerance to commonly used hospital biocides, and phenotypic expression of efflux pumps.

## 2 Material and Methods

This is a descriptive, retrospective study that analysed clinical isolates from hospitalized individuals in a period of 3 years (2021–2023). There were 31 clinical isolates of *T. asahii*, 39 clinical isolates of *C. albicans* and 24 isolates of other yeast species. The samples were collected by a clinical microbiology service in Juiz de Fora, Minas Gerais, Brazil, located in a private hospital with around 160 beds and includes specialized departments such as adult and neonatal ICUs, coronary and neurological units, surgical and medical wards, as well as outpatient care services.

Hospitalized patients of any age and sex with clinical manifestations and complementary findings (laboratory, histopathological and/or imaging) consistent with fungal infection, together with culture‐confirmed isolation of one of the study yeasts, were eligible for inclusion. To avoid duplicate data, only the first isolate from each patient was included, and subsequent isolates from the same individual were excluded. This study was conducted with the informed consent of all participants, in accordance with the project approved by the Ethics Committee for Research Involving Human Beings of the Federal University of Juiz de Fora, under CAAE 18611019.6.0000.5147 (approved on 12 December 2025).

### 2.1 Analysis of Medical Records

Clinical and epidemiological information was obtained through a systematic review of the electronic medical records of individuals with positive cultures for these isolates. The data collected included demographic characteristics (age and gender), inpatient unit, clinical specimen, antifungal therapy after diagnosis and clinical outcome. These data were compiled and organized using a standardized spreadsheet.

### 2.2 Assessment of the Integrity of Isolates

The samples from the collection were identified and preserved in sterile 2 mL vials with distilled water, also sterile, according to the method described by Diogo et al. [15]. Clinical isolates from the collection were cultured on Sabouraud Dextrose Chloramphenicol Agar (Neogen/Brazil) and incubated at 35°C for 48 h. Morphological characteristics were observed, and the Gram staining method was performed to assess growth, viability and purity of the isolates.

### 2.3 Identification of Yeasts

After incubation, the 94 isolates were identified by biochemical and physiological methods using the Vitek 2 system (bioMérieux/Marcy‐l'Étoile, France), according to the manufacturer's instructions. The reference strains *C. albicans* ATCC 14053, *C. parapsilosis* ATCC 22019, *C. tropicalis* ATCC 66029, *N. glabratus* (syn. *C. glabrata* ) ATCC 2001 and *T. asahii* ATCC 90039 served as the quality control standard, yielding a 99.9% match, thereby validating the identification process.

### 2.4 Antifungal Susceptibility Testing

The strains isolated and identified were assessed for their antimicrobial susceptibility profile following the CLSI (*Clinical and Laboratory Standard Institute*) guidelines [16]. The disk diffusion technique was used to evaluate the response of these isolates to fluconazole (25 μg), voriconazole (1 μg), caspofungin (5 μg) and amphotericin B (20 μg) (Liofilchem Diagnostic, Roseto degli Abruzzi (Te), Italy). Inhibition zones around the discs were measured after 24 h of growth at 35°C.

The interpretation of the inhibition zones for *T. asahii* was performed according to Pfaller et al. [17] for fluconazole and voriconazole, Menezes et al. [18] for amphotericin B, and the manufacturer's instructions (Liofilchem Diagnostic, Roseto degli Abruzzi (Te), Italy), and CLSI M60 2020 [19] for caspofungin. The interpretation of inhibition zones for *C. albicans* and other yeast species was performed according to the reference described in document CLSI M44 [16] and the criteria of the manufacturer's instructions (Liofilchem Diagnostic, Roseto degli Abruzzi (Te), Italy).

### 2.5 Assessment of Tolerance to Biocides

Tolerance to hospital‐use biocides was evaluated using an adaptation of the disk diffusion method described by the CLSI M44 [16].

Isolate suspensions adjusted to a 0.5 McFarland turbidity standard were evenly spread onto Mueller–Hinton agar (Kasvi, Pinhais, Brazil). Filter paper disks impregnated with 5 μL of sodium hypochlorite (1%, 1.5% and 2%) (Start, São Paulo, Brazil), 4.25% hydrogen peroxide (Rioquímica, São José do Rio Preto, Brazil), and 5% benzalkonium chloride (Êxodo Científica, São Paulo, Brazil) were then placed on the agar surface. Inhibition zones were measured after 24 h of incubation at 35°C. The biocides used were of commercial grade, stored in regular conditions and used within the validity periods. All the tests were performed with duplicates for all clinical isolates.

### 2.6 Evaluation of Phenotypic Efflux Pump Activity

The phenotypic efflux pump activity was assessed using ethidium bromide (EtBr) (Ludwig, Porto Alegre, Brazil), according to the method described by Cartwheel et al. (2011) [20] with modifications. The Mueller Hinton agar (Kasvi, Pinhais, Brazil) was prepared by adding ethidium bromide at concentrations of 0.5 μg/mL, 1.0 μg/mL, 1.5 μg/mL, 2.0 μg/mL and 2.5 μg/mL, and plates containing only the Mueller Hinton agar (Kasvi, Pinhais, Brazil) medium were used as the control for microbial growth. The colonies from this culture were inoculated to obtain a suspension in sterile saline solution (Sanobiol, Pouso Alegre, Brazil) on the 0.5 MacFarland turbidity scale and inoculated using a Steers replicator into the culture medium. Duplicates were made for each concentration and control. The plates were incubated for 24 h at 35°C, protected from light. After incubation, the plates were read under ultraviolet light, and the presence of fluorescence emitted by the samples was a negative indication of the action of the efflux pump.

### 2.7 Statistical Analysis

Descriptive statistical analysis was performed, including percentage, absolute frequency, range and mean values for individuals' age, in addition to the mean values of the inhibition halo diameters and their standard deviation obtained around the discs impregnated with biocides.

## 3 Results

The clinical and epidemiological parameters of the isolates are summarized in Table 1, highlighting the differences between *T. asahii*, *C. albicans* and other yeasts. Regarding the collection unit, isolates predominantly originated from intensive care units, particularly the general ICU, accounting for 51.3% of *C. albicans* isolates and 66.7% of other yeasts, whereas *T. asahii* was more frequently recovered from inpatient wards (29.1%) and the neurological ICU (35.5%).

**TABLE 1:** Clinical and epidemiological characteristics of individuals with positive cultures for clinically relevant yeasts (*n* = 94).

Clinical and epidemiological parameters	*Trichosporon asahii* (*n* = 31)	*Candida albicans* (*n* = 39)	Others yeast (*n* = 24)
Collection unit: *n* (%)			
Coronary unit	01 (3.2)	03 (7.7)	00 (0.0)
Impatient unit	09 (29.1)	05 (12.8)	03 (12.5)
Surgical centre	05 (16.1)	01 (2.6)	02 (8.3)
General ICU	03 (9.7)	20 (51.3)	16 (66.7)
Neurological ICU	11 (35.5)	10 (25.6)	03 (12.5)
Neonatal ICU	02 (6.4)	00 (0.0)	00 (0.0)
Clinical specimen: *n* (%)			
Bronchoalveolar lavage	10 (32.3)	00 (0.0)	01 (4.2)
Catheter tip	00 (0.0)	01 (2.6)	00 (0.0)
Sputum	00 (0.0)	01 (2.6)	00 (0.0)
Tracheal aspirate	02 (6.4)	16 (41.0)	11 (45.8)
Urine	19 (61.3)	21 (53.8)	12 (50.0)
Clinical outcome: *n* (%)			
Death	16 (51.6)	28 (71.8)	15 (62.5)
Hospital discharge	15 (48.4)	11 (28.2)	09 (37.5)
Use of antifungals after diagnosis: *n* (%)			
No	17 (54.8)	31 (79.5)	16 (66.7)
Yes	14 (45.2)	08 (20.5)	08 (33.3)
Yes—Combination therapy	06 (42.8)	00 (0.0)	02 (25.0)
Yes—Anidulafungin	00 (0.0)	01 (12.5)	01 (12.5)
Yes—Micafungin	00 (0.0)	01 (12.5)	00 (0.0)
Yes—Nystatin	01 (7.2)	01 (12.5)	02 (25.0)
Yes—Amphotericin B	00 (0.0)	03 (37.5)	02 (25.0)
Yes—Fluconazole	04 (28.6)	01 (12.5)	01 (12.5)
Yes—Itraconazole	03 (21.4)	01 (12.5)	00 (0.0)

Urine was the predominant specimen across all groups, accounting for 61.3% of *T. asahii* isolates, 53.8% of *C. albicans* isolates and 50.0% of other yeast isolates (Table 1).

Concerning clinical outcomes, high mortality rates were observed in all groups, with the highest frequency among *C. albicans* cases (71.8%) (Table 1).

Most individuals did not receive antifungal therapy after diagnosis, particularly those with *C. albicans* (79.5%), while 54.8% of individuals with *T. asahii* isolates remained untreated (Table 1).

**FIGURE 1:**
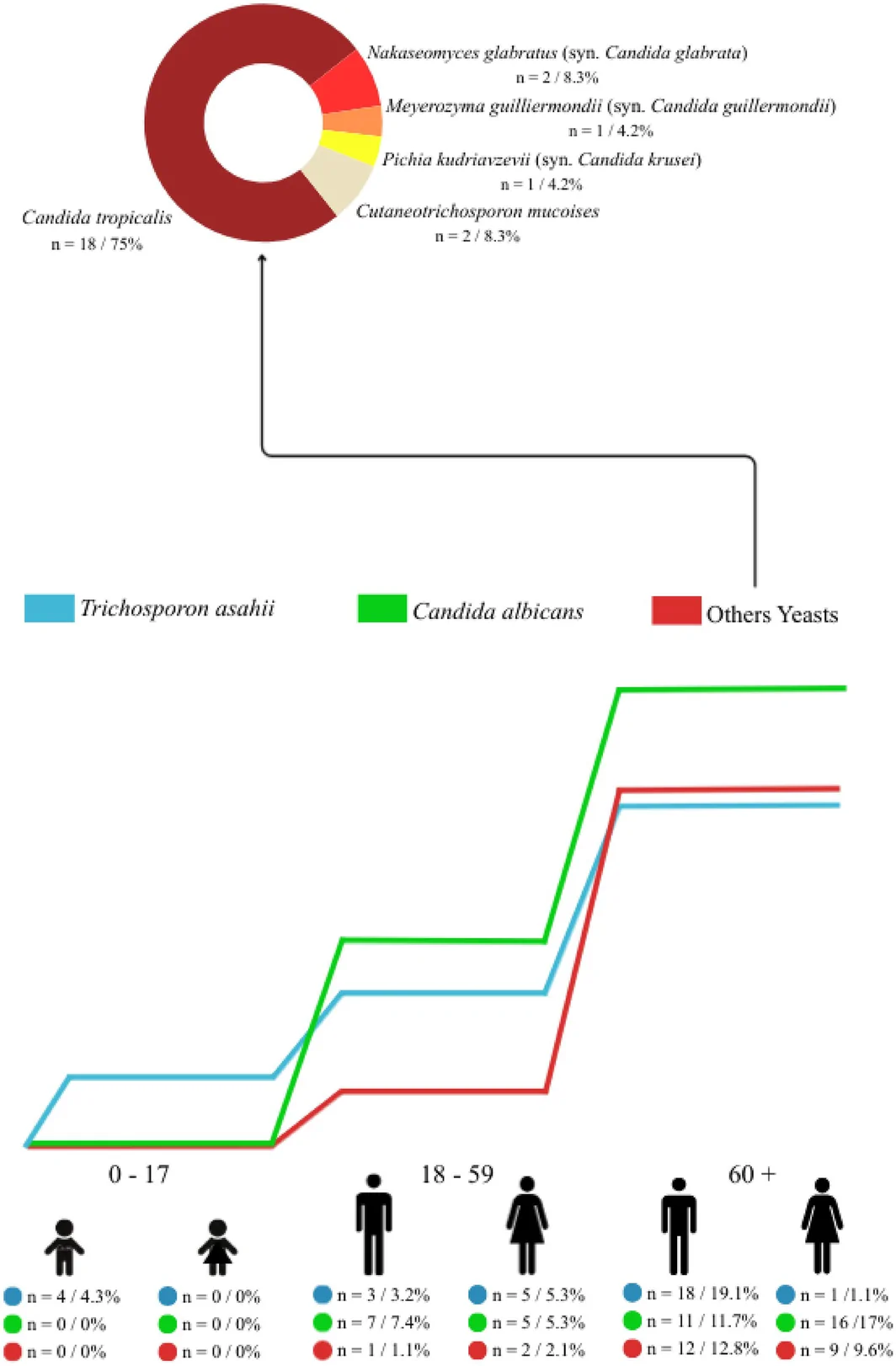
Age and sex distribution of participants according to fungal isolate (*n* = 94).

Analysis of medical records revealed a heterogeneous group with diverse characteristics, including a wide age distribution encompassing children, adults and elderly participants, as shown in Figure 1.

Twenty‐four samples were positive for other medically important yeasts, with *C. tropicalis* being the most common species, followed by *N. glabratus* (syn. *C. glabrata* ), *Cutaneotrichosporon mucoides*, *P. kudriavzevii* (syn. *C. krusei* ) and *Meyerozyma guilliermondii* (syn. *Candida guilliermondii* ) (Figure 1 and Table S1A,B).

**FIGURE 2:**
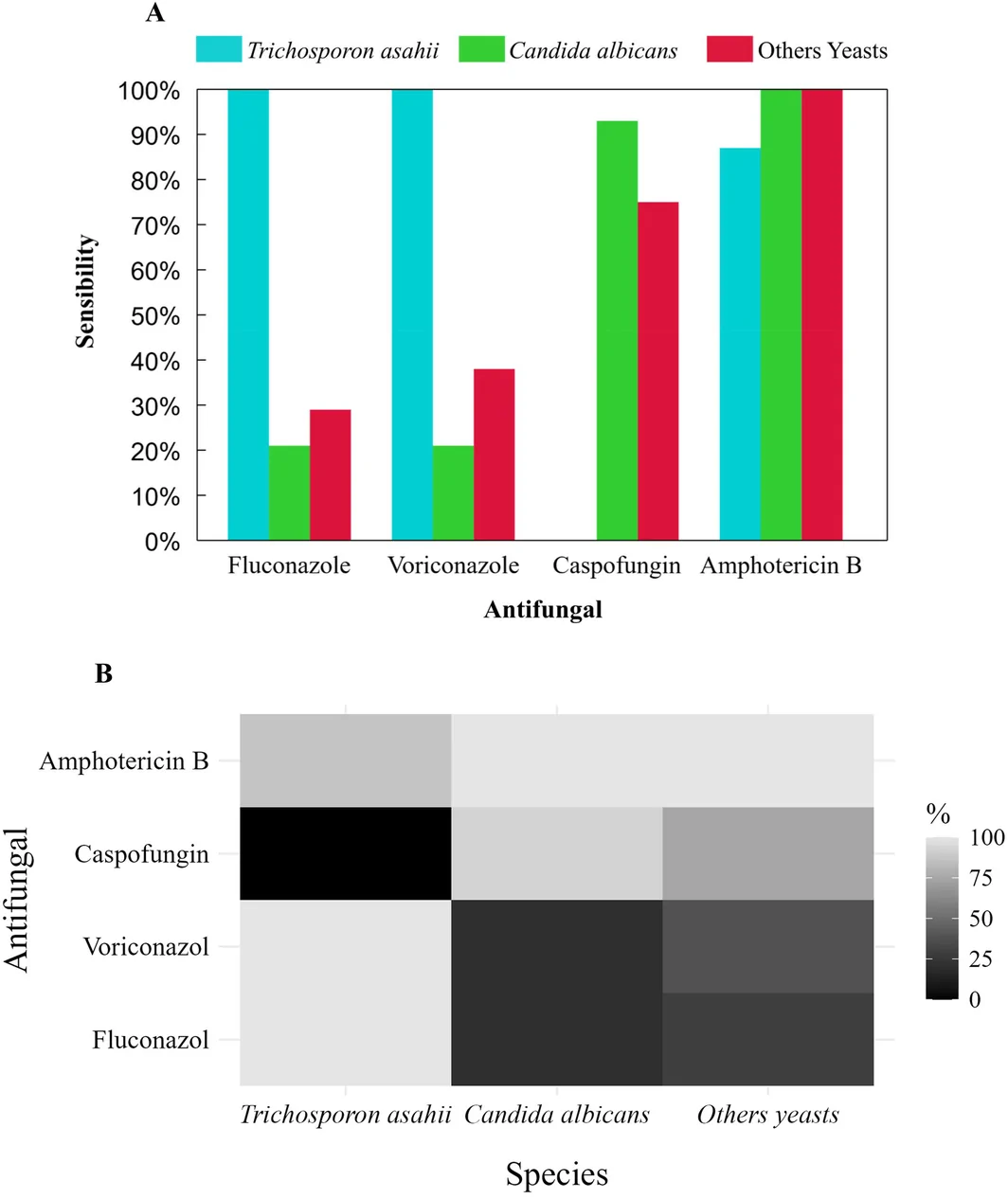
Antifungal susceptibility profiles among clinical yeast isolates (*n* = 94). (A) *T. asahii =* blue, *C. albicans* = green and other yeasts = red. (B) Higher susceptibility to the tested antifungal agents is represented by lighter grey tones, whereas lower susceptibility is indicated by darker tones.

The antifungal susceptibility test revealed that the *T. asahii* isolates were resistant to caspofungin, and 26/87% were susceptible to amphotericin B. In the group positive for *C. albicans* , all samples were susceptible to amphotericin B, whereas only 8/21% of the samples were susceptible to fluconazole and voriconazole, and 36/93% were susceptible to caspofungin. Among samples positive for other yeasts, all isolates were susceptible to amphotericin B; 18/75% were susceptible to caspofungin, 9/38% to voriconazole and 5/21% to fluconazole (Figure 2 and Table S1A,B).

**FIGURE 3:**
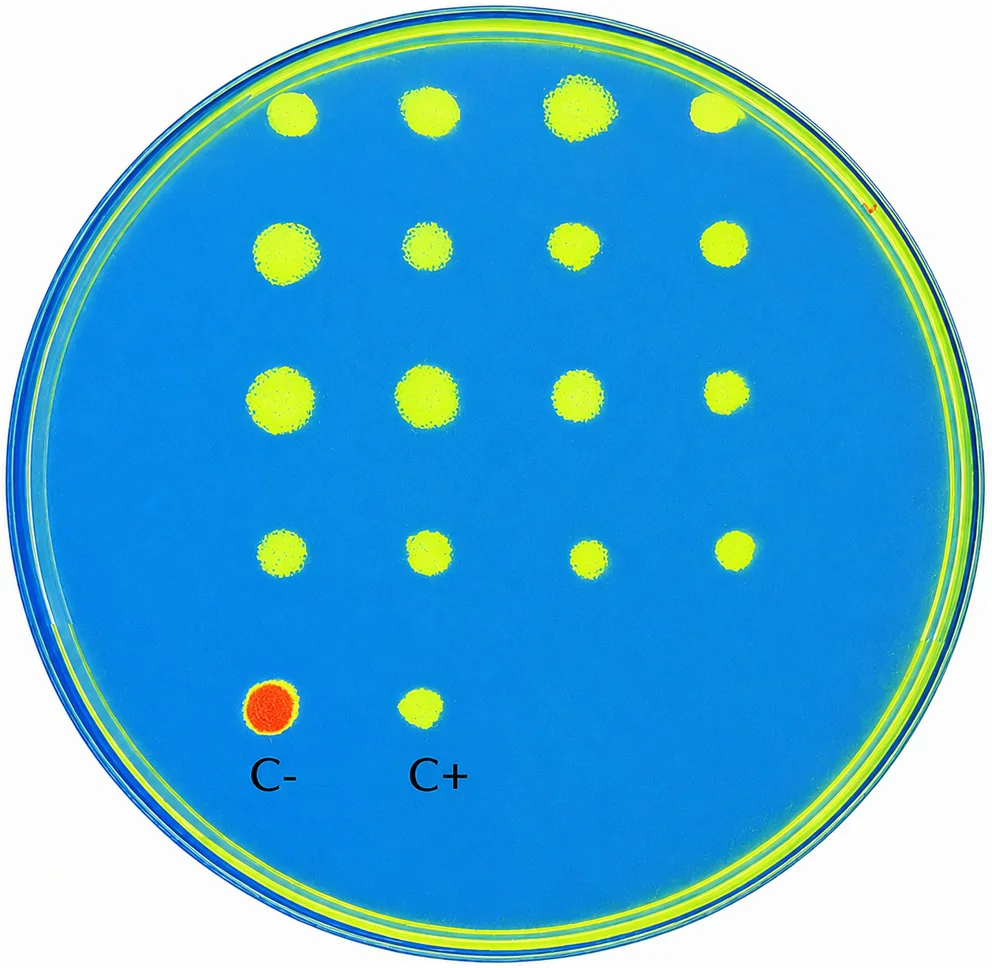
Efflux pump expression assessed by ethidium bromide (EtBr) assay. C+: Positive control: Green = Absence of fluorescence = positive for efflux pump. C−: Negative control: Red = Presence of fluorescence = negative for efflux pump. The remaining spots represent samples with absence of fluorescence and are therefore positive for efflux pump activity.

The results presented in Table 2 demonstrate a high frequency of isolates positive for phenotypic efflux pump activity, as assessed by exposure to different concentrations of ethidium bromide (EtBr) across all yeast groups analysed. Interpretation of efflux pump activity: Positive results correspond to isolates exhibiting the green fluorescence pattern (absence of intracellular fluorescence), whereas negative results correspond to isolates exhibiting the red fluorescence pattern (presence of intracellular fluorescence), as illustrated in Figure 3.

**TABLE 2:** Phenotypic expression of efflux pumps in *Trichosporon asahii*, *Candida albicans* and other yeasts at different ethidium bromide concentrations.

EtBr concentration	Isolates		
	*Trichosporon asahii* (*n* = 31)	*Candida albicans* (*n* = 39)	Others yeasts (*n* = 24)
0.5% (*n*/%)	31/100.0	32/82.1	19/79.2
1.0% (*n*/%)	28/90.3	37/94.8	24/100.0
1.5% (*n*/%)	31/100.0	36/92.3	21/87.5
2.0% (*n*/%)	31/100.0	37/94.8	20/83.3
2.5% (*n*/%)	31/100.0	33/84.6	18/75.0

For *T. asahii* (*n* = 31), a high proportion of positive isolates was observed at all tested concentrations, with 100% positivity for EtBr, and a slight reduction to 90.3% (28/31) at 1.0%. These findings indicate consistent efflux pump expression in this species, largely independent of the EtBr concentration evaluated.

In *C. albicans* (*n* = 39), most isolates were positive at all concentrations, with positivity rates ranging from 82.1% (32/39) to 94.8% (37/39). The highest frequencies were observed at 1.0% and 2.0% EtBr (94.8%), whereas the lowest was recorded at 0.5% (82.1%), suggesting moderate variability in the phenotypic expression of efflux pumps according to EtBr concentration.

Among the other yeasts (*n* = 24), the proportion of positive isolates ranged from 75.0% (18/24) to 100% (24/24). The highest positivity was observed at 1.0% EtBr, while lower frequencies were detected at 2.0% and 2.5%, indicating potential heterogeneity in efflux pump activity within this group.

Overall, these results demonstrate a high prevalence of efflux pump expressions among *T. asahii*, *C. albicans* and other yeast isolates, supporting the role of this mechanism as an important contributor to tolerance against chemical compounds, including biocides (and Table S1A,B).

Table 3 summarizes the biocide tolerance results as mean inhibition of halo diameters (mm). At 1% sodium hypochlorite, mean halos were 10.8 mm for *T. asahii*, 12.6 mm for *C. albicans* and 12.3 mm for other yeasts. Increasing concentration to 1.5% led to larger halos (12.8 mm, 14.6 mm and 15.1 mm, respectively). The greatest inhibitory effect was observed at 2%, with mean diameters of 16.0 mm for *T. asahii*, 17.8 mm for *C. albicans* and 18.0 mm for the remaining yeasts.

**TABLE 3:** Tolerance of *Trichosporon asahii*, *Candida albicans* and other yeasts to different biocides, evaluated by the average diameter of the inhibition halo.

Biocide	Average inhibition halo diameter (mm) (±SD)		
	*Trichosporon asahii* (*n* = 31)	*Candida albicans* (*n* = 39)	Others yeasts (*n* = 24)
Sodium hypochlorite 1%	10.8 (±3.54)	12.6 (±2.43)	12.3 (±1.98)
Sodium hypochlorite 1.5%	12.8 (±4.68)	14.6 (±3.31)	15.1 (±2.92)
Sodium hypochlorite 2%	16.0 (±5.83)	17.8 (±4.15)	18.0 (±3.86)

*Note:* Smaller inhibition zone diameters indicate greater tolerance of yeasts to the evaluated biocides.

Abbreviation: SD, Standard deviation.

In contrast, the analysed yeasts showed tolerance to 4.25% hydrogen peroxide and 5% benzalkonium chloride, with absence of inhibition halo and low variability, regardless of the species tested.

## 4 Discussion

A global shift in the epidemiological profile has been observed, with an increasing incidence of infections caused by non‐albicans *Candida* species, including *Nakaseomyces glabratus* (syn. *C. glabrata* ) and *Pichia kudriavzevii* (syn. *C. krusei* ), which are often associated with reduced susceptibility or intrinsic resistance to azoles, posing additional therapeutic challenges [21]. In parallel, species of the genus *Trichosporon* have gained relevance as agents of invasive infections, frequently showing limited response to amphotericin B and echinocandins. In this context, our findings corroborate these data, highlighting the clinical importance of these yeasts in healthcare settings, particularly among patients in critical condition [21, 22].

The distribution of isolates across hospital units revealed high frequency in intensive care settings, consistent with the recognized vulnerability of these patients. The high frequency in these sectors likely reflects the complexity of clinical management, including prolonged hospitalization, use of invasive devices, exposure to broad‐spectrum antimicrobials, and the presence of comorbidities [7, 8, 21]. Urine was the main clinical specimen in all groups, possibly associated with urinary catheter use and the ability of these yeasts to form biofilms and colonize both biotic and abiotic surfaces [7, 23].

This study demonstrated an increased frequency of cases with advancing age, with 71.3% of isolates obtained from individuals over 60 years old, predominantly male, possibly associated with immunosenescence. The higher occurrence among males represents a relevant finding that remains underexplored in the literature, particularly regarding potential differences in the resident mycobiota between men and women [24, 25].

The therapeutic arsenal available for the treatment of systemic fungal infections is limited. The finding that 89.4% of isolates were resistant to at least one antifungal agent is concerning. Then, azole antifungals, such as fluconazole and voriconazole, represent a central therapeutic option for yeast infections due to their broad‐spectrum activity [26, 27]. The high resistance rates observed for fluconazole (51.1%), followed by voriconazole (49.0%) in this study, discourage the empirical use of these agents as first‐line antifungal therapy.

In contrast, amphotericin B remains active against many isolates in most reports. A study conducted in Anhui hospitals reported that *Candida* species predominated among clinical isolates, and many isolates of *Candida* showed decreased susceptibility to itraconazole, fluconazole and voriconazole, whereas amphotericin B retained high in vitro activity [28]. Similarly, our study demonstrated a comparable susceptibility profile. Isolates of *C. albicans* showed reduced susceptibility to fluconazole and voriconazole, with only 21% of the samples classified as susceptible. All *C. albicans* isolates were susceptible to amphotericin B.

Among the *C. tropicalis* isolates analysed, a worrying profile of antifungal resistance was observed, particularly in relation to caspofungin and amphotericin B. All isolates were resistant to amphotericin B (100.0%), while a high proportion were also resistant to caspofungin (83.3%), suggesting possible compromise of the efficacy of two therapeutic classes widely used in the management of candidemia. This corroborates a recent study with *C. tropicalis* isolates, which observed high resistance rates of 60% to fluconazole, 51.4% to amphotericin B and 25.7% to caspofungin [29]. This resistance profile may have relevant clinical implications, since clinical outcomes showed a high mortality rate among infected individuals, with 66.7% progressing to death, while only 33.3% were discharged from the hospital. Although multiple clinical and epidemiological factors may influence prognosis, the high frequency of antifungal resistance observed in these *C. tropicalis* isolates may have contributed to the severity of the cases. Early antifungal therapy is critical, as delays can significantly increase mortality [30].

Although six *T. asahii* isolates exhibited in vitro resistance to amphotericin B, which is considered a last‐line antifungal agent [26, 27], therapeutic strategies in such cases may include adjustments in dosage and/or treatment duration with this polyene, as well as the possible use of combination therapy with other antifungal agents. In contrast, Xia and colleagues (2022) reported that isolates of *Trichosporon* exhibited reduced susceptibility to itraconazole, fluconazole and voriconazole at varying levels, while remaining comparatively more susceptible in vitro to amphotericin B [28].

In contrast to *Candida* spp., *Trichosporon* spp. requires a distinct therapeutic interpretation. The reduced in vitro activity of caspofungin observed against *T. asahii* in this study is consistent with the known low susceptibility of this genus to echinocandins [9]. Azoles remain more relevant agents in this context, and all *T. asahii* isolates evaluated here were susceptible to fluconazole and voriconazole [31]. Although six isolates showed reduced in vitro susceptibility to amphotericin B, which is considered a last‐line antifungal agent [26, 27], therapeutic strategies in such cases may include adjustments in dosage and/or treatment duration with this polyene, as well as the possible use of combination therapy with other antifungal agents. Variability in amphotericin B activity against *Trichosporon* has also been described in the literature. Xia et al. [28], for example, reported reduced susceptibility to itraconazole, fluconazole and voriconazole in some *Trichosporon* isolates, whereas amphotericin B showed comparatively greater in vitro activity. Together, these findings reinforce that trichosporonosis should not be managed according to the same treatment framework used for invasive candidiasis and that antifungal choice should be guided by species identification and susceptibility data whenever possible.

A total of 68% of the participants in this study did not receive antifungal therapy, even after laboratory confirmation of yeast infection. Among these individuals, approximately 61% progressed to death, highlighting the severity of the studied population and suggesting possible delays, limitations, or failures in prescribing antifungal treatment. This finding is consistent with the literature, as a study involving *Candida* species reported that 46.8% of patients with laboratory‐confirmed fungal infection also did not receive antifungal therapy [32], possibly due to the predominance of urinary isolates, since candiduria may represent colonization, contamination, or true infection and should be interpreted in conjunction with the patient's clinical condition [32, 33]. These findings reinforce the importance of careful clinical assessment and timely initiation of antifungal therapy, always based on the correlation between laboratory findings and the patient's clinical status.

Among participants who received antifungal treatment and presented *C. albicans* isolates (*n* = 8), five patients received an azole derivative. However, only 21% of the isolates were susceptible to azole derivatives, indicating that this therapeutic approach may not represent the most appropriate treatment choice in these cases. On the other hand, among participants with *Trichosporon* isolates, 14 patients received azole derivatives, the most frequently prescribed antifungal class in this group. All isolates were susceptible to fluconazole and voriconazole, indicating an appropriate therapeutic option in these cases. These findings highlight the importance of accurate laboratory diagnosis of yeast infections in association with a targeted therapeutic approach, allowing antifungal treatment to be better aligned with the susceptibility profile of the microorganism.

The phenotypic expression of efflux pumps was assessed using an exploratory in vitro screening assay, which revealed elevated levels in all groups, particularly in *T. asahii*, suggesting an adaptive mechanism aimed at expelling diverse compounds, including antifungals and disinfectants, thereby favouring survival and persistence in hospital environments [34, 35].

Among the evaluated biocides, sodium hypochlorite demonstrated the greatest inhibitory activity, especially at higher concentrations. In contrast, yeasts showed high tolerance to 4.25% hydrogen peroxide and 5% benzalkonium chloride, which may contribute to environmental persistence when these compounds are used. When selecting biocides, it is essential to consider not only their efficacy but also safety, the type of surface to be disinfected, and cost [13]. Sodium hypochlorite is commonly employed as a disinfectant in clinical settings; however, its application is constrained by various factors. Its high reactivity can result in the corrosion of medical equipment and sensitive surfaces. Furthermore, its volatility and the release of chlorine gas can lead to respiratory and eye irritation, presenting health risks to both staff and individuals [14].

The main biocides used in healthcare settings include ethyl alcohols, hydrogen peroxide, quaternary ammonium compounds, and active chlorine release agents, each with different mechanisms of action and practical applications [36]. The recommendations for their application in clinical and hospital environments define specific concentrations and instructions for use, playing a vital role in infection control and maintaining health safety [13].

This study has some limitations that should be considered when interpreting the results. It was conducted at a single‐centre, which may limit the generalizability of the findings to other epidemiological settings. Additionally, the absence of molecular analyses to characterize genes associated with antifungal resistance and efflux pump expression restricts a deeper understanding of the genetic mechanisms underlying the observed phenotypes. Nevertheless, the results provide relevant and original contributions by describing, in a clinical context, antifungal resistance profiles and biocide tolerance patterns among medically important yeasts.

## 5 Conclusion

This study highlights the clinical and epidemiological relevance of yeasts such as *T. asahii*, *C. albicans* and others among hospitalized individuals, reinforcing their role as important opportunistic pathogens in healthcare settings. These findings underscore the need for accurate laboratory diagnosis, continuous education of healthcare professionals, ongoing monitoring of antifungal susceptibility profiles, and the implementation of effective infection prevention and control strategies, including the rational selection of biocides, to improve the management of fungal infections in hospital environments.

## Author Contributions

**Letícia Rebello Machado:** investigation, methodology, visualization, writing – review and editing. **Victor Quinet de Andrade Bastos:** conceptualization, investigation, resources. **Lucas Quinet de Andrade Bastos:** conceptualization, investigation, resources. **Vanessa Cordeiro Dias:** conceptualization, funding acquisition, investigation, writing – original draft, writing – review and editing, project administration, supervision, resources, data curation. **Ricardo Villela Bastos:** conceptualization, resources, investigation. **André Netto Bastos:** conceptualization, investigation, resources, methodology. **Claudio Galuppo Diniz:** conceptualization, methodology, resources. **Yasmim Passos Lima:** investigation, methodology, visualization, writing – review and editing. **Vania Lúcia da Silva:** resources, methodology, conceptualization.

## Funding

This work was supported by the Coordination for the Improvement of Higher Education Personnel (CAPES), Graduate Program in Biological Sciences (PPGCBIO), Research Support Foundation of the State of Minas Gerais (FAPEMIG—APQ‐00925‐25), Federal University of Juiz de Fora (UFJF) and Cortes Villela Laboratory.

## Ethics Statement

This study was reviewed and approved by the Research Ethics Committee of the Federal University of Juiz de Fora (CEP/UFJF), Brazil. All methods were carried out in accordance with relevant institutional guidelines and regulations.

## Supporting information


**Table S1:** (A) Clinical and epidemiological aspects of individuals with positive cultures for clinically relevant yeasts (*n* = 24); (B) Antifungal susceptibility profile, biocide tolerance and efflux pump expression among yeast isolates (*n* = 24).

## Data Availability

All data generated or analysed during this study are included in this published article and its Supporting Information.
