# Supplementary material for: Antifungal Susceptibility Profile and Biocide Tolerance in Clinically Relevant Yeasts: Implications for Therapy and Hospital Infection Control
Source: Mycoses. 2026 Sep 4;69(9):e70216. doi: 10.1111/myc.70216 (PMC13543762; doi:10.1111/myc.70216)
Supplement: Supplementary file 1 [file MYC-69-e70216-s001.docx]

**Supplementary Table S1A and B. (A)** Clinical and epidemiological aspects of individuals with positive cultures for clinically relevant yeasts (n = 24); (B) Antifungal susceptibility profile, biocide tolerance, and efflux pump expression among yeast isolates (n = 24)

| **Parameters** | | ***Candida tropicalis*** | ***Nakaseomyces glabratus (syn. Candida glabrata)*** | ***Meyerozyma guilliermondii (syn. Candida guilliermondii)*** | ***Pichia kudriavzevii (syn. Candida krusei)*** | ***Cutaneotrichosporon mucoides (syn. Trichosporon mucoides)*** |
| --- | --- | --- | --- | --- | --- | --- |
|  | | **n = 18** | **n = 02** | **n = 01** | **n = 01** | **n = 2** |
| A) Clinical and epidemiological aspects of individuals with positive cultures for clinically relevant yeasts (n = 24) | | | | | | |
| Gender: n (%) | |  |  |  |  |  |
|  | Female | 7 (38.8) | 02 (100.0) | 00 (0.0) | 01 (100.0) | 01 (50.0) |
|  | Male | 11 (61.2) | 00 (0.0) | 01 (100.0) | 00 (0.0) | 01 (50.0) |
| Age: n (%) | | |  |  |  |  |
|  | < 17 years | 00 (0.0) | 00 (0.0) | 00 (0.0) | 00 (0.0) | 00 (0.0) |
|  | 18 – 59 years | 02 (11.2) | 00 (0.0) | 00 (0.0) | 00 (0.0) | 01 (50.0) |
|  | > 60 years | 16 (88.8) | 02 (100.0) | 01 (100.0) | 01 (100.0) | 01 (50.0) |
| Collection unit: n (%) | | |  |  |  |  |
|  | Coronary unit | 00 (0.0) | 00 (0.0) | 00 (0.0) | 00 (0.0) | 00 (0.0) |
|  | Impatient unit | 02 (11.1) | 01 (50.0) | 00 (0.0) | 00 (0.0) | 00 (0.0) |
|  | Surgical center | 01 (5.6) | 00 (0.0) | 00 (0.0) | 00 (0.0) | 01 (50.0) |
|  | General ICU | 13 (72.2) | 01 (50.0) | 00 (0.0) | 01 (100.0) | 01 (50.0) |
|  | Neurological ICU | 02 (11.1) | 00 (0.0) | 01 (100.0) | 00 (0.0) | 00 (0.0) |
|  | Neonatal ICU | 00 (0.0) | 00 (0.0) | 00 (0.0) | 00 (0.0) | 00 (0.0) |
| Clinical specimen: n (%) | | |  |  |  |  |
|  | Bronchoalveolar lavage | 00 (0.0) | 00 (0.0) | 00 (0.0) | 00 (0.0) | 01 (50.0) |
|  | Catheter tip | 00 (0.0) | 00 (0.0) | 00 (0.0) | 00 (0.0) | 00 (0.0) |
|  | Sputum | 00 (0.0) | 00 (0.0) | 00 (0.0) | 00 (0.0) | 00 (0.0) |
|  | Tracheal aspirate | 09 (50.0) | 00 (0.0) | 00 (0.0) | 01 (100.0) | 01 (50.0) |
|  | Urine | 09 (50.0) | 02 (100.0) | 01 (100.0) | 00 (0.0) | 00 (0.0) |
| Clinical outcome: n (%) | | |  |  |  |  |
|  | Death | 12 (66.7) | 01 (50.0) | 00 (0.0) | 01 (100.0) | 01 (50.0) |
|  | Hospital discharge | 06 (33.3) | 01 (50.0) | 01 (100.0) | 00 (0.0) | 01 (50.0) |
| B) Antifungal susceptibility profile, biocide tolerance, and efflux pump expression among yeast isolates (n = 24) | | | | | | |
| Use of antifungals after diagnosis: n (%) | | | |  |  |  |
|  | No | 13 (72.2) | 00 (0.0) | 00 (0.0) | 01 (100.0) | 02 (100.0) |
|  | Yes | 05 (27.8) | 02 (100.0) | 01 (100.0) | 00 (0.0) | 00 (0.0) |
|  | Yes - Combination therapy | 02 (40.0) | 00 (0.0) | 00 (0.0) | 00 (0.0) | 00 (0.0) |
|  | Yes - Anidulafungin | 01 (20.0) | 00 (0.0) | 00 (0.0) | 00 (0.0) | 00 (0.0) |
|  | Yes - Micafungin | 00 (0.0) | 00 (0.0) | 00 (0.0) | 00 (0.0) | 00 (0.0) |
|  | Yes- Nystatin | 01 (20.0) | 00 (0.0) | 01 (100.0) | 00 (0.0) | 00 (0.0) |
|  | Yes - Amphotericin B | 01 (20.0) | 01 (50.0) | 00 (0.0) | 00 (0.0) | 00 (0.0) |
|  | Yes - Fluconazole | 00 (0.0) | 01 (50.0) | 00 (0.0) | 00 (0.0) | 00 (0.0) |
|  | Yes - Itraconazole | 00 (0.0) | 00 (0.0) | 00 (0.0) | 00 (0.0) | 00 (0.0) |
| Antifungal resistance: n (%) | | |  |  |  |  |
|  | Fluconazole | 05 (27.8) | 00 (0.0) | 00 (0.0) | 00 (0.0) | 02 (100.0) |
|  | Voriconazole | 05 (27.8) | 01 (50.0) | 00 (0.0) | 01 (100.0) | 02 (100.0) |
|  | Caspofungin | 15 (83.3) | 02 (100.0) | 01 (100.0) | 00 (0.0) | 00 (0.0) |
|  | Amphotericin B | 18 (100.0) | 02 (100.0) | 01 (100.0) | 01 (100.0) | 02 (100.0) |
| Biocides tolerance: mean (mm) | | |  |  |  |  |
|  | Sodium hypochlorite 1% | 12.1 mm | 12.1 mm | 13.5 mm | 14.5 mm | 13.5 mm |
|  | Sodium hypochlorite 1.5% | 15.3 mm | 18.0 mm | 21.0 mm | 18.5 mm | 13.2 mm |
|  | Sodium hypochlorite 2% | 18.2 mm | 21.8 mm | 22.5 mm | 24.5 mm | 15.8 mm |
|  | Hydrogen peroxide 4.25% | 6.0 mm | 6.0 mm | 6.0 mm | 6.0 mm | 6.3 mm |
|  | Benzalkonium chloride 5% | 6.0 mm | 6.0 mm | 6.0 mm | 6.0 mm | 6.3 mm |
| Efflux pump (EtBr concentration): n (%) positive | | | |  |  |  |
|  | 0.5% | 15 (83.3) | 02 (100.0) | 00 (0.0) | 00 (0.0) | 02 (100.0) |
|  | 1.0% | 18 (100.0) | 02 (100.0) | 01 (100.0) | 01 (100.0) | 02 (100.0) |
|  | 1.5% | 15 (83.3) | 02 (100.0) | 01 (100.0) | 01 (100.0) | 02 (100.0) |
|  | 2.0% | 14 (77.7) | 02 (100.0) | 01 (100.0) | 01 (100.0) | 02 (100.0) |
|  | 2.5% | 12 (66.6) | 02 (100.0) | 01 (100.0) | 01 (100.0) | 02 (100.0) |
